# Benefits and harms of exercise therapy in people with multimorbidity: A systematic review and meta-analysis of randomised controlled trials

**DOI:** 10.1016/j.arr.2020.101166

**Published:** 2020-09-05

**Authors:** Alessio Bricca, Lasse K. Harris, Madalina Jäger, Susan M. Smith, Carsten B. Juhl, Søren T. Skou

**Affiliations:** a Research Unit for Musculoskeletal Function and Physiotherapy, Department of Sports Science and Clinical Biomechanics, University of Southern Denmark, 5230, Odense M, Denmark; b Department of Physiotherapy and Occupational Therapy, Nœstved-Slagelse-Ringsted Hospitals, Region Zealand, 4200, Slagelse, Denmark; c HRB Centre for Primary Care Research, Department of General Practice, Royal College of Surgeons in Ireland (RCSI), Dublin, Ireland; d Department of Physiotherapy and Occupational Therapy, University Hospital of Copenhagen Herlev and Gentofte, Copenhagen, Denmark

**Keywords:** Multimorbidity, Exercise, Quality of life, Physical function, Depression, Anxiety

## Abstract

**Objectives:**

To investigate the benefits and harms of exercise therapy on physical and psychosocial health in people with multimorbidity.

**Design:**

Systematic review of randomised controlled trials (RCTs). Data sources MEDLINE, EMBASE, CENTRAL and CINAHL from 1990 to April 20th, 2020 and Cochrane reviews on the effect of exercise therapy for each of the aforementioned conditions, reference lists of the included studies, the WHO registry and citation tracking on included studies in Web of Science.

**Eligibility criteria for study selection:**

RCTs investigating the benefit of exercise therapy in people with multi-morbidity, defined as two or more of the following conditions: osteoarthritis (of the knee or hip), hypertension, type 2 diabetes, depression, heart failure, ischemic heart disease, and chronic obstructive pulmonary disease on at least one of the following outcomes: Health-related quality of life (HRQoL), physical function, depression or anxiety.

**Summary and quality of the evidence:**

Meta-analyses using a random-effects model to assess the benefit of exercise therapy and the risk of non-serious and serious adverse events according to the Food and Drug Administration definition. Meta-regression analyses to investigate the impact of pre-specified mediators of effect estimates. Cochrane ‘Risk of Bias Tool’ 2.0 and the GRADE assessment to evaluate the overall quality of evidence.

**Results:**

Twenty-three RCTs with 3363 people, testing an exercise therapy intervention (mean duration 13.0 weeks, SD 4.0) showed that exercise therapy improved HRQoL (standardised mean difference (SMD) 0.37, 95 % CI 0.14 to 0.61) and objectively measured physical function (SMD 0.33, 95 % CI 0.17 to 0.49), and reduced depression symptoms (SMD -0.80, 95 % CI -1.21 to -0.40) and anxiety symptoms (SMD -0.49, 95 % CI -0.99 to 0.01). Exercise therapy was not associated with an increased risk of non-serious adverse events (risk ratio 0.96, 95 % CI 0.53-1.76). By contrast, exercise therapy was associated with a reduced risk of serious adverse events (risk ratio 0.62, 95 % CI 0.49 to 0.78). Meta-regression showed that increasing age was associated with lower effect sizes for HRQoL and greater baseline depression severity was associated with greater reduction of depression symptoms. The overall quality of evidence for all the outcomes was downgraded to low, mainly due to risk of bias, inconsistency and indirectness.

**Conclusions:**

Exercise therapy appears to be safe and to have a beneficial effect on physical and psychosocial health in people with multimorbidity. Although the evidence supporting this was of low quality, it highlights the potential of exercise therapy in the management and care of this population.

## 1 Introduction

Multimorbidity, the coexistence of two or more chronic conditions, is as a *priority for global health research* (Anon., 2018). Compared to people living with only one chronic condition, people with multimorbidity are more likely to die prematurely, to be admitted to and have an increased length of stay at the hospital, (Menotti et al., 2001; Vogeli et al., 2007) in addition to having poorer physical and psychosocial health, higher intake of multiple drugs and increased health care utilization (Bayliss et al., 2004; Fortin et al., 2006, 2004). The increasing burden and complexity of multimorbidity challenge current standards of care, which focus on single-disease management approaches rather than individualised care (Pefoyo et al., 2015; Sinnott et al., 2013). One approach to deal with the complexity of multimorbidity is to focus on specific combination of conditions, linked by physiological factors and risk factors (Smith et al., 2018, 2013).

Osteoarthritis of the knee or hip, hypertension, type 2 diabetes, depression, heart failure, ischemic heart disease, and chronic obstructive pulmonary disease are among the leading causes of global disability, affect hundreds of millions of people around the world, and often co-exist (Global Burden of Disease Study 2013 Collaborators, 2015). As an example, two out of three people with osteoarthritis have one or more other chronic conditions (Wesseling et al., 2013), with heart failure, ischemic heart disease, hypertension, type 2 diabetes, depression and chronic obstructive pulmonary disease being some of the most common (Reeuwijk et al., 2010). These conditions share a common risk factor (physical inactivity) and pathogenesis (systemic low-grade inflammation) which may trigger a cascade of reactions resulting in the development of a ‘vicious cycle’ of chronic diseases and poor outcomes (Gleeson et al., 2011; Pedersen and Saltin, 2015).

Several systematic reviews investigating the effect of exercise therapy have demonstrated the safety and benefits of exercise therapy on each of the aforementioned individual conditions, (Bartels et al., 2016; Cornelissen and Smart, 2013; Fransen et al., 2015; Long et al., 2019; Puhan et al., 2016) and clinical guidelines recommend exercise therapy as the cornerstone of treatment (National Institute for Health and Care Excellence, 2013). On the contrary, the few existing multimorbidity guidelines, summarised in a systematic review (Muth et al., 2019), encourage a healthy lifestyle, including regular physical activity, but do not include specific recommendations for exercise therapy, (Muth et al., 2019) which is among the top 10 research priorities for people with multimorbidity (Parker et al., 2019). Moreover, the interventions for people with multimorbidity evaluated so far have shown minor and negligible effects on important outcomes for people with multimorbidity such as health-related quality of life and function (Salisbury et al., 2018; Smith et al., 2016). One of the key features of exercise therapy is its anti-inflammatory effects at cellular, tissue and organ level as well as its positive psychosocial and physiological effects such as increase in muscle strength, improved blood pressure regulation and insulin sensitivity (Gleeson et al., 2011; Pedersen and Saltin, 2015). The positive effects of exercise therapy may hence disrupt the ‘vicious cycle’ of systemic inflammation and improve physical and psychosocial health in people with multimorbidity. This highlights the importance of investigating whether exercise therapy can be recommended as a cornerstone treatment also for people with multimorbidity. No previously published systematic reviews exist on this topic.

We therefore performed a systematic review of randomised controlled trials (RCTs) on the benefits and harms of exercise therapy in people with multimorbidity defined as at least two of the following conditions: osteoarthritis of the knee or hip, hypertension, type 2 diabetes, depression, heart failure, ischemic heart disease, and chronic obstructive pulmonary disease on health-related quality of life, physical function, depression and anxiety.

## 2 Methods

This systematic review was guided by the recommendations for performing systematic reviews in the Cochrane Handbook (Higgins et al., 2019) and the reporting was performed according to the Preferred Reporting Items for Systematic Reviews and Meta-analyses (PRISMA) guidelines (Moher et al., 2009).

### 2.1 Protocol

The protocol for this systematic review has been published, (Bricca et al., 2020) and it was registered at PROSPERO (CRD42020150628). The statistical analysis plan was made publicly available on the Open Science Framework website (Foster and Deardorff, 2017) (https://osf.io/38fcg/) before the title and abstract (TIAB) screening phase was initiated.

### 2.2 Eligibility criteria

#### 2.2.1 Study design and participants

RCTs published in peer-reviewed journals. We included studies, with adult (>18 years old), reporting at least 80 % of participants with two or more of the following conditions: osteoarthritis of the knee or hip, heart failure, ischemic heart disease, hypertension (systolic blood pressure >90 and diastolic blood pressure >140), type 2 diabetes mellitus, chronic obstructive pulmonary disease and depression as defined by the studies or calculated based on baseline participants characteristics. This pragmatic approach was pre-specified and adopted in an effort to capture all the studies which included people with multimorbidity, given the expected inconsistency of reporting of the conditions across trials.

#### 2.2.2 Intervention

We included exercise therapy interventions with or without additional pharmacotherapy or other adjuvant interventions (e.g. weight loss), based in all settings, including at least 80 % of participants with multimorbidity as defined in our study. Exercise therapy is defined as ‘a regimen or plan of physical activities designed and prescribed for specific therapeutic goals with the purpose of restoring normal physical function or to reduce symptoms caused by diseases or injuries’ (Caspersen et al., 1985).

We excluded interventions not delivering a structured exercise therapy programme such as providing only a pedometer or booklet to the participants without a specific plan for general physical activity.

#### 2.2.3 Comparator

We included studies comparing exercise therapy interventions to usual/standard care (e.g. counselling from their health care provider), at the time the trial was conducted, and comparator groups non-exposed such as wait-and-see and placebo treatments.

#### 2.2.4 Outcomes

We included studies assessing at least one of the following outcomes:

- Physical health: Objectively measured and self-reported physical function (e.g. 6-minute walking test, 36-item Short-Form Health Survey (SF-36))
- Psychosocial health: Health-related quality of life (e.g. EQ-5D questionnaire), depression symptoms and anxiety symptoms (e.g. Hospital Anxiety and Depression Scale),
- Adverse events according to the FDA definition (Anon., 2020) that is any unfavourable and unintended sign, symptom or disease temporally associated with the use of a medicinal (investigational) product whether or not considered related to the medicinal (investigational) product and grouped in serious adverse events such as death, hospitalization, disability or permanent damage, and non-serious adverse events such as pain, falls and fatigue.

The rationale for including these outcomes is based on a consensus study which identified outcomes for multimorbidity intervention studies (Smith et al., 2018) and the fact that they are generic and widely used across the conditions of interest. Additionally, the choice of these outcomes was supported by the patient partner of MOBILIZE who were made aware of the systematic review and outcome measures included.

### 2.3 Literature search

We obtained information from searching MEDLINE via PubMed, EMBASE via Ovid, CINAHL (including preCINAHL) via EBSCO and the CENTRAL with no restriction on language. We only included RCTs published from 1990 since the reporting as well as the treatment of multimorbidity has changed substantially within the last years, up to October 12^th^, 2019. The search was repeated for the period from October 2019 to April 20^th^, 2020 in the same databases to identify additional studies published before manuscript submission. We also screened the reference lists of the latest Cochrane reviews investigating the effect of exercise therapy on the following conditions: osteoarthritis, hypertension, type 2 diabetes, depression, heart failure, ischemic heart disease, chronic obstructive pulmonary disease, the reference lists of included RCTs. Furthermore, we screened for completed trials in The World Health Organization’s International Clinical Trials Registry Platform (ICTRP) http://apps.who.int/trialsearch/ which comprises the 16 primary registries of the WHO registry network and ClinicalTrials. gov, and searched Web of Science (WoS) for studies citing the RCTs included in this systematic review (citations tracking).

### 2.4 Search method and study selection

The search strategy was developed for MEDLINE (https://osf.io/84vzn/) and was customised for EMBASE, CINAHL and CENTRAL. All terms were searched both as keywords (Mesh) and as text words in title and abstract, if possible. To identify RCTs, we used the Cochrane sensitive search strategy for identifying RCTs. The results of the literature search were uploaded to EndNote X9.3.1. Two reviewers (AB and LKH) independently screened titles and abstracts and all studies deemed eligible by at least one of the reviewers were checked independently in full text by the same two reviewers. Disagreement between the reviewers about inclusion of individual studies was discussed until consensus was reached. When disagreement persisted, a third reviewer (CBJ) was contacted to resolve the disagreement. We checked whether multiple reports from the same study were published by juxtaposing author names, treatment comparisons, sample sizes and outcomes. When multiple reports of the same study provided different study characteristics (e.g. number of participants and presence of one or more chronic conditions) we used the primary publication. We recorded the reasons for excluding full text RCTs.

### 2.5 Data collection

We used a modified version of the Cochrane Collaboration data collection form for intervention reviews: RCTs only (Higgins et al., 2019). When a study had two intervention groups with two different exercise therapy interventions (A and B) and one usual care comparator group (C), we split the comparator group in two groups of smaller sample size and compared A and B versus C, and reported the results as two separate study comparisons. This procedure is in accordance with the Cochrane handbook (Higgins et al., 2019).

We extracted the following data, from the follow-up immediately after the end of the intervention and the follow-ups closest to 12 months, for continuous outcomes (number of participants, mean and standard deviation, standard error or 95 % Confidence Interval) and for categorical outcomes, we extracted adverse events (cases and total number of participants):

- Trial characteristics: location of the trial (e.g. Country), number of patients allocated to the exercise and comparator groups, respectively, number of patients in the intention to treat (ITT) and per protocol analysis, in the intervention and comparator groups, respectively.
- Participant characteristics: Age, % female, body mass index (BMI), socioeconomic status (SES) (studies were labelled as low socioeconomic status when the majority of the participants were described as having low education levels, low income, being unemployed or sample otherwise labelled as ‘low SES’), baseline severity and diagnosis of the conditions, and number, type and frequency of other conditions.
- Intervention and comparator characteristics: Components of intervention (e.g. exercise therapy + education), type of intervention/comparator interventions (aerobic, neuromuscular, strengthening or a combination of those), frequency of the sessions (times per week), intensity of the session (% of maximum pulse, or % of 1 Repetition Maximum), primary mode of delivery (individual, group, self-help), setting (home-based, hospital-based, or clinic/facility/rehabilitation centres based), duration of the interventions (in weeks), supervision (yes, no or a combination), tailoring (intervention developed according to guidelines and individual patients’ needs), adherence to intervention (number of sessions attended out of the total number of planned sessions).
- Outcome characteristics: time points assessed and the magnitude of objectively and subjectively measured changes (e.g. change in health-related quality of life, number of adverse events in the intervention and comparator groups). To avoid multiplicity, we used a hierarchy of selection rules for the outcomes. We prioritised data extraction of outcome measures important for the participants (Smith et al., 2018) and generic over disease-specific measures (Bricca et al., 2020). For objectively measured physical function, we prioritised: 1) the 6-minute walking test, 2) Incremental Shuttle Walking Test, 3) any other outcome measure related to daily function (e.g. Chair stand test). For self-reported physical function, we prioritised outcomes in the following order: 1) the 36-item Short-Form Health Survey (SF-36) Physical Function subscale, 2) the SF-36 Role Function subscale, 3) any other self-reported measure of physical function. For health-related quality of life outcomes, we prioritised: 1) the EQ-5D questionnaire, 2) any other health-related quality of life questionnaires, 3) disease-specific health related quality of life questionnaires (e.g. The Minnesota living with heart failure questionnaire). For depression symptoms, we prioritised: 1) The Beck Depression Inventory (BDI), 2) any other depression questionnaire (e.g. the Hospital Anxiety and Depression Scale (HADS depression). For anxiety symptoms, we prioritised: 1) State Trait Anxiety Inventory questionnaire, 2) any other anxiety questionnaire (e.g. HADS anxiety).

If the data could not be extracted from the published studies, we emailed the corresponding author of the study with a checklist including the data we aimed to obtain. If the email we sent bounced back, we contacted the second author and so forth. After three days, we sent a reminder including the last author of the paper. After seven days of the first email, we re-sent the email to the corresponding and last author. A second reminder followed ten days after the first email. We considered the data as missing after not receiving any communication from the authors fifteen days after we sent the first email.

### 2.6 Risk of bias assessment and overall evaluation of the quality of the evidence

Two reviewers (AB and LKH) independently assessed the risk of bias of the included studies using the Cochrane ‘Risk of Bias Tool 2.0’ (Higgins et al., 2019). Bias was assessed in five distinct domains: Bias arising from the randomisation process or lack of allocation concealment, Bias

due to deviations from intended interventions, Bias due to missing outcome data, Bias in measurement of the outcome or delivery of the intervention (blinding), Bias in selection of the reported result. Within each domain, the two reviewers answered one or more signalling questions (e.g. Was the allocation sequence random? Were participants aware of their assigned intervention during the trial?) which lead to judgments of “low risk of bias,” “some concerns,” or “high risk of bias“. The judgments within each domain lead to an overall risk-of-bias judgment for the outcome being assessed (Higgins et al., 2019). The overall quality (or certainty) of evidence for the estimates were evaluated using the GRADE (Grading of Recommendations Assessment, Development and Evaluation) approach (Schünemann et al., 2013). GRADE is a systematic approach to rate the certainty of evidence across studies for specific outcomes. It is based on five domains that involve the methodological flaws of the studies (i.e., risk of bias), the heterogeneity of results across studies (i.e., inconsistency), the generalisability of the findings to the target population (i.e., indirectness), the precision of the estimates and the risk of publication bias.

### 2.7 Synthesis of results

#### 2.7.1 Main analyses

We performed meta-analyses using a random-effects model as heterogeneity was expected in participant, intervention and outcome characteristics. For physical function, we performed two separate meta-analyses for objectively and self-reported outcomes, respectively. Standardised mean differences (SMD) with 95 % CIs were calculated for outcome measures of continuous data and adjusted to Hedges g. The magnitude of the effect size of the pooled SMD was interpreted as 0.2 representing a small effect, 0.5 a moderate effect, and 0.8 a large effect (Cohen, 1988). Effect estimates above 0.5 were considered clinically relevant (Cohen, 1988). The relative risk (RR) of serious and non-serious adverse events was estimated separately as the rate of adverse events in the intervention group divided by the rate of adverse events in the control group. A random effects meta-analysis was applied to estimate the overall RR of adverse events in the exercise therapy groups compared with comparator groups (Higgins et al., 2019). Heterogeneity was examined as between-study variance and calculated as the I-squared statistic measuring the proportion of variation in the combined estimates due to between study variance. An I-squared value of 0% indicates no inconsistency between the results of individual trials, and an I-squared value of 100 % indicates maximal inconsistency. Meta-analyses were performed in STATA (16.1) using the ‘meta’ command.

#### 2.7.2 Analysis of subgroups and meta-regression analyses

For the pre-planned sub-group analyses of studies reporting objectively measured physical function with the 6-minute walk test (6MWT), we estimated the weighted mean difference (WMD) between the intervention and comparator groups. Meta-regression analyses were also performed to identify factors (covariates) which predicted better health outcome when more than ten studies provided data for the covariates of interest, in accordance with the Cochrane Handbook recommendations (Higgins et al., 2019). Relevant study-level covariates were defined as those able to decrease the between-study variance Tau-square (and thus inconsistency measured as the I-squared statistic) (Higgins et al., 2019). Pre-identified factors were extracted based on a systematic screening of the latest Cochrane systematic reviews investigating the effect of exercise therapy on each of the individual conditions of interest and input from members of the study team. These factors were pre-specified and made publicly available prior to the title and abstract screening (https://osf.io/zx9vw/), and are related to participant, intervention and outcome measure characteristics and listed in the ‘data collection’ above. Additionally, we investigated the impact of risk of bias on the estimates for the outcomes of interest by classifying studies at “low risk of bias”, “some concerns”, or “high risk of bias” according to the Cochrane Risk of Bias tool 2.0.

#### 2.7.3 Patients’ involvement

The MOBILIZE project is committed to patient involvement and has so far included patients living with multimorbidity in all aspects of the decision-making process in the project. Their experiences, needs and preferences play an important role in the development of a novel intervention (Collaborate level on the IAP2 Spectrum of Public Participation).

## 3 Results

### 3.1 Study selection and characteristics

**Fig. 1:**
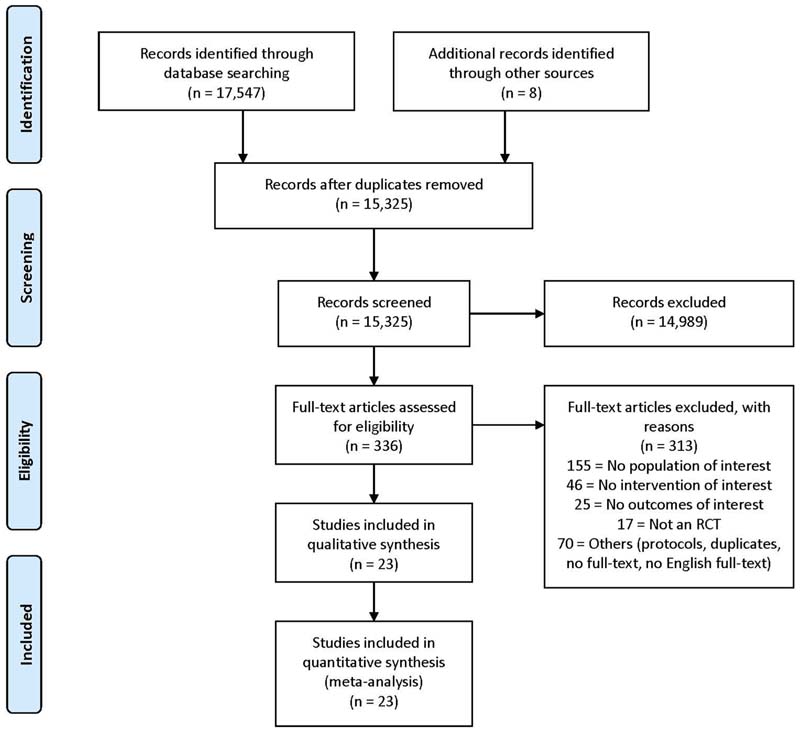
Flow chart of the study selection process. RCT = Randomised controlled trial.

The literature search identified a total of 17,547 unique publications, of which 336 individual RCTs were identified and full texts screened for potential eligibility. We ultimately included 23 RCTs from 24 papers involving 41 study comparisons (Fig. 1). One study was reported in two different papers (Edelmann et al., 2011; Nolte et al., 2015). We included them all since they report different outcome measures in different papers and counted them as one study. The number of study comparisons exceed the number of studies because when several intervention groups were included in a study, the between-group difference was reported for each possible comparison, at different follow-ups.

The included studies were conducted across 17 countries, including Europe, (Edelmann et al., 2011; Nolte et al., 2015; Pibernik-Okanovic et al., 2015; Oerkild et al., 2012; Hinrichs et al., 2016; Koukouvou et al., 2004; Bernocchi et al., 2018a; Campo et al., 2020; Asa et al., 2012; Kulcu et al., 2007; Rodriguez-Manas et al., 2019) USA (Blumenthal et al., 2012a; de Groot et al., 2019; Gary et al., 2012, 2010; Gary et al., 2004; Gretebeck et al., 2019; Schneider et al., 2016; Blumenthal et al., 2012b), Australia (Leung et al., 2013), and Asia (Leung et al., 2019; Keihani et al., 2014; Abdelbasset et al., 2019; Soliman and Abdelbasset, 2019). The characteristics of the included studies are reported in Table 1.

### 3.2 Participants

The most common conditions reported in the 23 RCTs (3363 people) The most common conditions reported in the 23 RCTs (3363 people) were heart failure in 16 studies, depression and type 2 diabetes in 15 studies each, hypertension in 14 studies, chronic obstructive pulmonary disease in 6 studies and osteoarthritis of the knee or hip in 4 studies each. The number of conditions reported per study varied from two to seven and the most common combinations of conditions were heart failure and depression, (Koukouvou et al., 2004; Kulcu et al., 2007; Blumenthal et al., 2012a; Gary et al., 2010; Blumenthal et al., 2012b; Keihani et al., 2014; Abdelbasset et al., 2019; Bernocchi et al., 2018b) type 2 diabetes and depression (Pibernik-Okanovic et al., 2015; de Groot et al., 2019; Schneider et al., 2016), and hypertension and type 2 diabetes (Rodriguez-Manas et al., 2019; Gretebeck et al., 2019; Leung et al., 2019) (Table 1).

### 3.3 Intervention and comparator groups

Aerobic exercise was the most commonly applied type of exercise therapy (n = 11), (Oerkild et al., 2012; Asa et al., 2012; Kulcu et al., 2007; Blumenthal et al., 2012a; de Groot et al., 2019; Gary et al., 2010, 2004; Blumenthal et al., 2012b; Keihani et al., 2014; Abdelbasset et al., 2019; Soliman and Abdelbasset, 2019) followed by exercise programmes combining aerobic, strengthening, balance and flexibility exercises (n = 8), (Edelmann et al., 2011; Nolte et al., 2015; Pibernik-Okanovic et al., 2015; Hinrichs et al., 2016; Koukouvou et al., 2004; Campo et al., 2020; Gary et al., 2012; Schneider et al., 2016; Bernocchi et al., 2018b), and Tai Chi (n = 2) (Leung et al., 2013, 2019), or resistance training alone (n = 2), (Rodriguez-Manas et al., 2019; Gretebeck et al., 2019). All the included trials provided total or partial supervision of the exercise sessions. Adherence to the exercise therapy sessions varied widely from 51 % to 94 % of the planned exercise sessions. Interventions were delivered individually (Edelmann et al., 2011; Nolte et al., 2015; Kulcu et al., 2007; Rodriguez-Manas et al., 2019; Keihani et al., 2014), in group (Pibernik-Okanovic et al., 2015; Koukouvou et al., 2004; Asa et al., 2012; Blumenthal et al., 2012a; de Groot et al., 2019; Gretebeck et al., 2019; Schneider et al., 2016; Blumenthal et al., 2012b; Leung et al., 2013, 2019), or as self-help (Oerkild et al., 2012; Hinrichs et al., 2016; Campo et al., 2020; Gary et al., 2012, 2010, 2004; Abdelbasset et al., 2019; Soliman and Abdelbasset, 2019; Bernocchi et al., 2018b), (i.e. participants were recommended to exercise by themselves with a structured programme) with or without partial supervision. Exercise interventions were performed at home (45 %), at a fitness centre or clinical medical centre (45 %) or at a hospital (10 %). Comparator groups varied widely and included usual care, medication, cognitive behavioural therapy, education about health conditions, consultations with General Practitioners and stretching and flexibility exercises (Table 1).

### 3.4 Outcomes

Health-related quality of life was reported in thirteen RCTs (Oerkild et al., 2012; Hinrichs et al., 2016; Koukouvou et al., 2004; Campo et al., 2020; Asa et al., 2012; Kulcu et al., 2007; de Groot et al., 2019; Gary et al., 2012, 2010; Gary et al., 2004; Leung et al., 2013, 2019; Bernocchi et al., 2018b). Physical function was reported in fifteen RCTs of which thirteen assessed physical function objectively (Edelmann et al., 2011; Oerkild et al., 2012; Hinrichs et al., 2016; Campo et al., 2020; Asa et al., 2012; Rodriguez-Manas et al., 2019; de Groot et al., 2019; Gary et al., 2012, 2010; Gary et al., 2004; Gretebeck et al., 2019; Leung et al., 2013; Bernocchi et al., 2018b), and two subjectively (Leung et al., 2019; Keihani et al., 2014). Depression symptoms and anxiety symptoms were reported in fifteen, (Oerkild et al., 2012; Koukouvou et al., 2004; Asa et al., 2012; Kulcu et al., 2007; Blumenthal et al., 2012a; Gary et al., 2012, 2012, 2010; Gary et al., 2004; Schneider et al., 2016; Leung et al., 2013; Keihani et al., 2014; Abdelbasset et al., 2019; Soliman and Abdelbasset, 2019) and six studies (Oerkild et al., 2012; Koukouvou et al., 2004; Asa et al., 2012; Kulcu et al., 2007; Leung et al., 2013; Keihani et al., 2014), respectively. Characteristics of the outcome measures are reported in Table 1.

### 3.5 Effect of exercise therapy on health-related quality of life

**Fig. 2:**
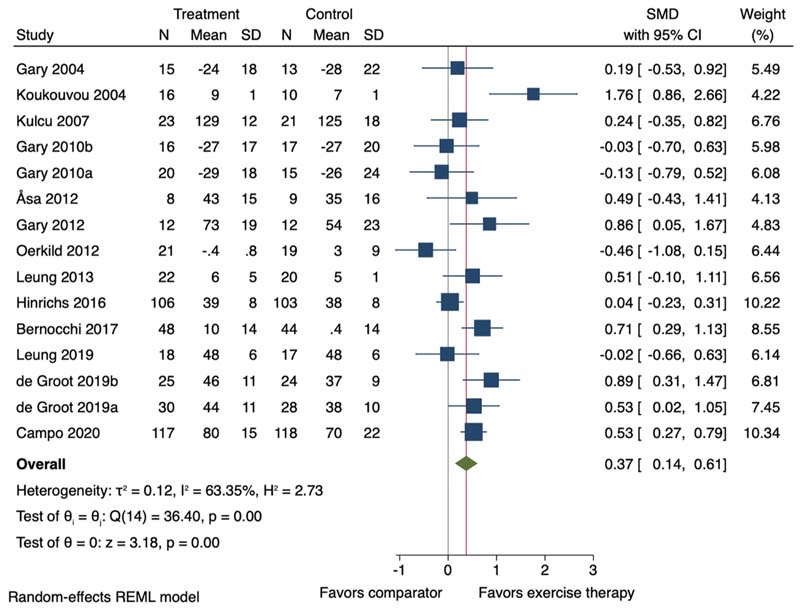
Forest plot for the effect of exercise therapy compared to a non-exercise therapy comparator group on health-related quality of life. SMD = Standardised Mean Difference; 95 % CI = 95 % Confidence Interval. ^a, b^=two separate study comparisons.

Thirteen of the 23 studies (including 15 study comparisons) were included in the meta-analysis. Exercise therapy, average duration 13.3 weeks (SD 4.8), promoted a small improvement in HRQoL (SMD 0.37, 95 % CI 0.14 to 0.61; I^2^ = 63.35 %) (Fig. 2). Meta-regressions indicated that increasing age was associated with lower effect sizes (slope -0.03, 95 % CI -0.05 to -0.01), suggesting that for each additional year in the study, the effect size was reduced by 0.03 SDs (Supplementary Table 1).

When including follow-up closest to 12-month post randomisation, mean 39.7-week (SD 14.2), exercise therapy promoted a small effect on HRQoL (mean n = 5, SMD 0.35, 95 %CI 0.18 to 0.52; I^2^ = 22.41 %) (Supplementary Fig. 1).

### 3.6 Effect of exercise therapy on objectively measured physical function

**Fig. 3:**
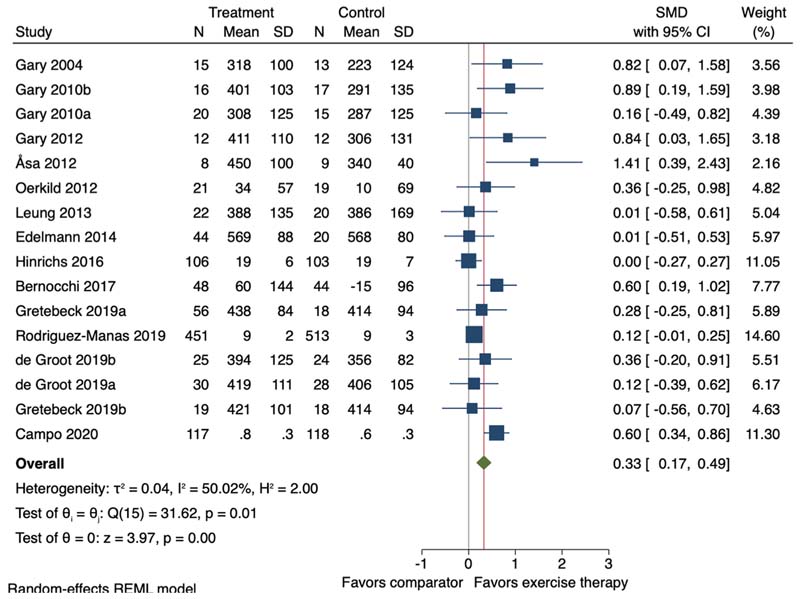
Forest plot for the effect of exercise therapy compared to a non-exercise therapy comparator group on objectively measured physical function. SMD = Standardised Mean Difference; 95 % CI = 95 % Confidence Interval. ^a,b^=two separate study comparisons.

**Fig. 4:**
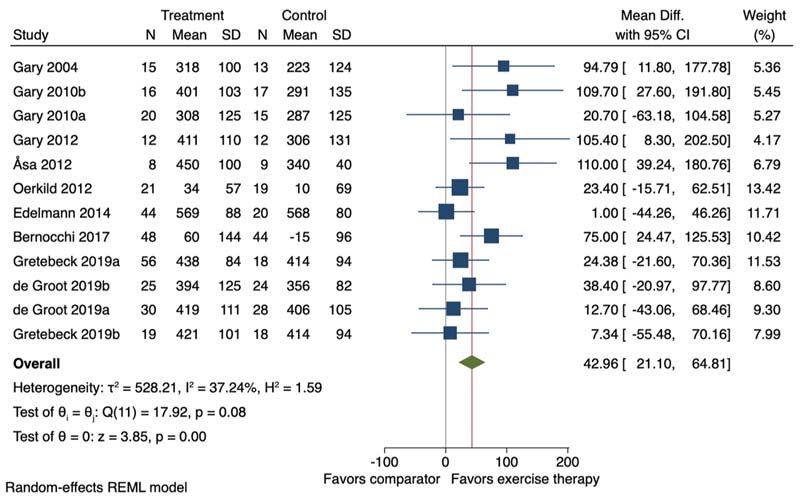
Forest plot for the effect of exercise therapy compared to a non-exercise therapy comparator group on the 6MWT. 95 % CI = 95 % Confidence Interval. ^a,b^=two separate study comparisons.

Thirteen of the 23 studies (including 16 study comparisons) were included in the meta-analysis. Exercise therapy, average duration 12.8 weeks (SD 3.7), promoted a small improvement on objectively measured physical function (SMD 0.33, 95 % CI 0.17 to 0.49, I^2^ = 50.02 %) (Fig. 3). In studies assessing objectively measured physical function using the 6MWT (n = 10) exercise therapy promoted an improvement of 42.96 m (95 % CI 21.10–64.81; I^2^ = 37.24 %) (Fig. 4).

When including follow-up closest to 12-month post randomisation, mean 33.5-week (SD 15.4), exercise therapy promoted a small improvement in objectively measured physical function (n = 6, SMD 0.38, 95 %CI 0.15 to 0.61; I^2^ = 60.08 %) (Supplementary Fig. 2).

### 3.7 Effect of exercise therapy on self-reported physical function

**Fig. 5:**
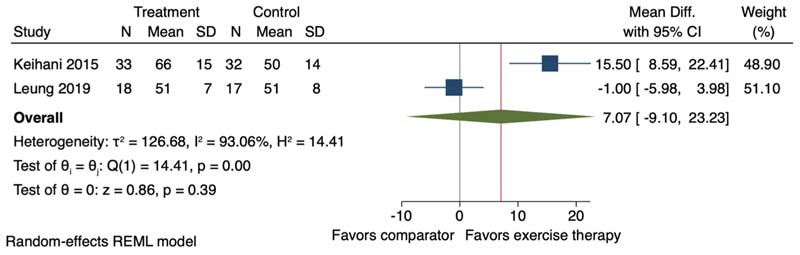
Forest plot for the effect of exercise therapy compared to a non-exercise therapy comparator group on self-reported physical function. 95 % CI = 95 % Confidence Interval. ^a,b^=two separate study comparisons.

Two of the 23 studies were included in this meta-analysis. Exercise therapy, average duration 10.0 weeks (SD 2.0), had no effect on self-reported physical function, assessed with SF-12 and SF-36 physical function subscale (MD 7.07, 95 %CI -9.10–23.23; I^2^ = 93.03 %) (Fig. 5).

### 3.8 Effect of exercise therapy on depression symptoms

**Fig. 6:**
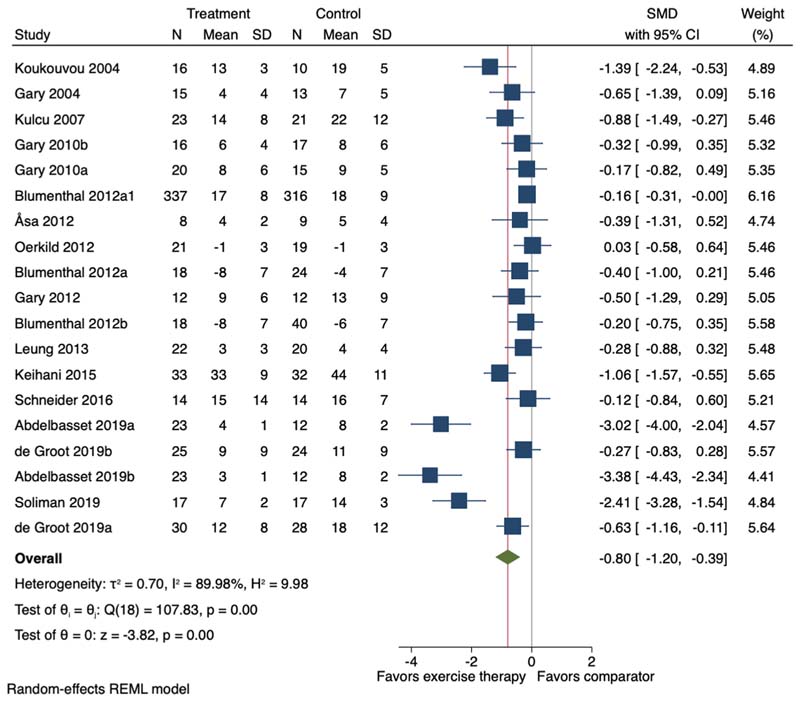
Forest plot for the effect of exercise therapy compared to a non-exercise therapy comparator group on depression symptoms. SMD = Standardised Mean Difference; 95 % CI = 95 % Confidence Interval. ^a,b^=two separate study comparisons.

Fifteen of the 23 studies (including 19 study comparisons) were included in the meta-analysis. Exercise therapy, average duration 13.2 weeks (SD 4.3), promoted a large reduction in depression symptoms (SMD -0.80, 95 % CI -1.20 to -0.39; I^2^ = 89.98 %) (Fig. 6).

Meta-regression showed that studies including participants with higher levels of depression were associated with higher reduction in depression symptoms (SMD -0,04, 95 % CI -0.07 to -0.02) Supplementary Fig. 3), suggesting that for each additional increase in depression score, the effect size increases by 0.04 SDs (Supplementary Table 1).

When including follow-up closest to 12-month post randomisation, mean 39.7-week (SD 14.5), exercise therapy had no effect on depression (n = 6; SMD -0.08, 95 % CI -0.23 to 0.07; I^2^ = 9.58 %) (Supplementary Fig. 4).

### 3.9 Effect of exercise therapy on anxiety symptoms

**Fig. 7:**
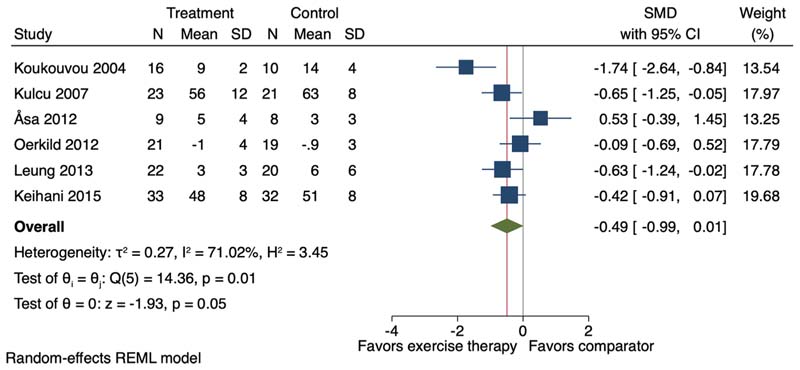
Forest plot for the effect of exercise therapy compared to a non-exercise therapy comparator group on anxiety symptoms. SMD = Standardised Mean Difference; 95 % CI = 95 % Confidence Interval.

Six of the 24 studies (including six study comparisons) were included in the meta-analysis investigating the effect of exercise therapy on anxiety. Exercise therapy (mean 13.2-week, SD 13.4) appeared to have a moderate effect on anxiety (SMD -0.49, 95 % CI -0.99 to 0.01; I^2^ = 71.02 %) (Fig. 7). Only one study assessed long term effects (52-week follow-up) of exercise on anxiety symptoms reporting no effect (SMD 0.03, 95 % CI -0.58 to -0.64).

### 3.10 Adverse events after exercise therapy in people with multimorbidity

**Fig. 8:**
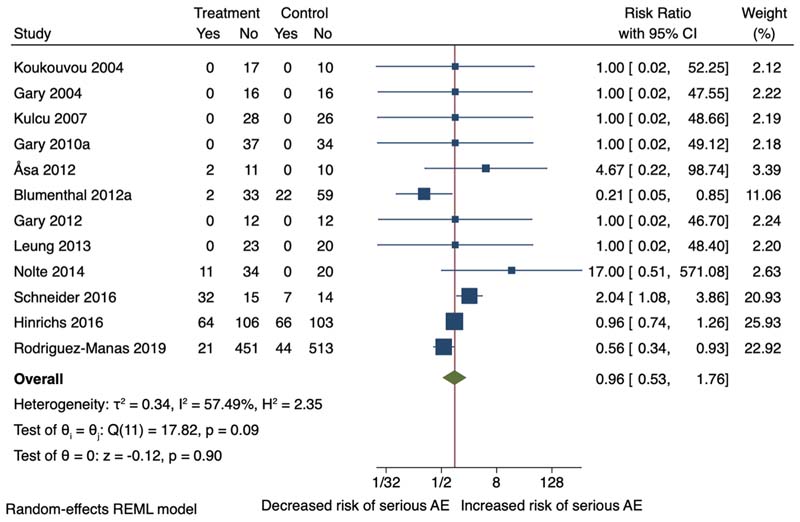
Forest plot non-serious adverse events.

**Fig. 9:**
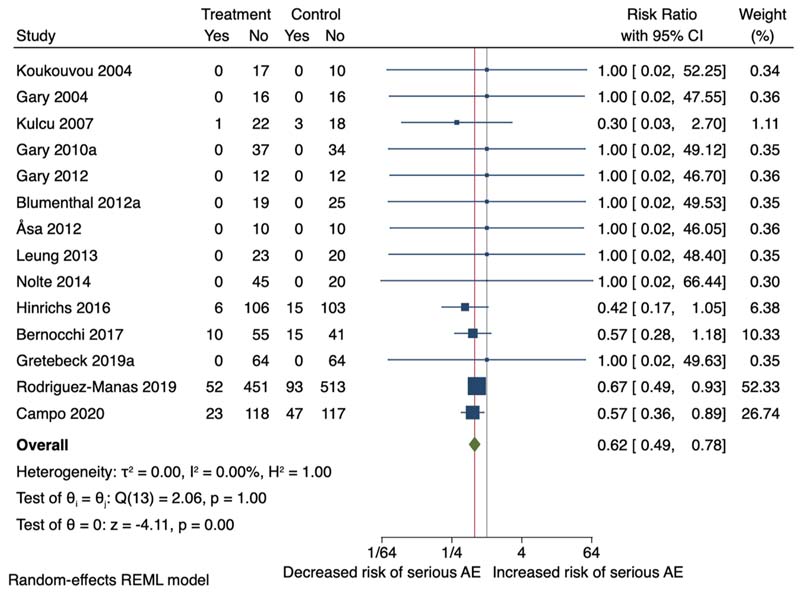
Forest plot serious adverse events.

Fourteen of the 23 RCTs reported non-serious adverse events data, of which thirteen were included in separate meta-analyses for serious and non-serious adverse events. One study (de Groot et al., 2019) was not included in the meta-analysis due to insufficient data and this study reported no difference in adverse events in the intervention vs the comparator group. The non-serious adverse events reported were knee, arm or back pain, falls, arrythmias, syncope, fatigue and sexual problems. The serious adverse events reported were hospitalisation, pneumonia, cardiac decompression and uncontrolled ventricular arrythmia, sepsis, and extreme fatigue. Meta-analysis showed no difference of non-serious adverse events (RR 0.96, 95 %CI 0.53–1.76; I^2^ = 57.49 %) (Fig. 8). However, exercise therapy reduced the risk of serious adverse events, including hospitalisation, death, pneumonia and cardia disorders (RR 0.62, 95 %CI 0.49 to 0.78; I^2^ = 0.00 %) between the intervention and comparator groups (Fig. 9).

### 3.11 Risk of bias assessment and overall evaluation of the quality of the evidence

Forty-three percent of the included studies were deemed as ‘low’ risk of bias, while 57 % as ‘some concerns’ or ‘high’ risk of bias (Supplementary Fig. 5). The majority of the RCTs applied a proper randomisation process and reported and assessed the outcomes of interest correctly. Also, there was no clear sign of publication bias in the funnel plots of each meta-analysis (Supplementary 6). However, all the studies were judged as ‘some concerns’ for the ‘measurement of the outcome’ item since it is not possible to blind participants to exercise interventions. Sensitivity analyses indicated that studies judged as ‘some concerns’ or ‘high’ risk of bias reported more favourable results in favour of the intervention groups than studies judged as ‘low’ risk of bias for the outcomes depression. We therefore downgraded the quality of evidence for depression due to risk of bias. The overall quality of the evidence assessed using GRADE, including reasons for downgrading the quality of the evidence, is summarised in Table 2.

## 4 Discussion

This systematic review included 23 RCTs using various types of exercise therapy administered in different settings and including more than 3300 people with multimorbidity from 17 countries. Although based on low quality of evidence, we found that exercise therapy is safe and effective to improve physical and psychosocial health. The greatest benefits from exercise therapy were reported for depression, anxiety, health-related quality of life and objectively measured physical function. While exercise therapy characteristics had no impact on physical and psychosocial health, younger people may expect greater improvements in health-related quality of life, and people with higher baseline depression may expect greater reduction in depression following an exercise therapy intervention.

### 4.1 Results in context

The exercise therapy types used included aerobic exercise (47 %), strengthening exercise (9%), a combination of these (35 %), and Tai Chi (9%), and were performed at home (45 %), at a fitness centres or clinical medical centre based (45 %) or at a hospital (10 %). The greatest benefits from exercise therapy were observed for depression, followed by anxiety, health-related quality of life and objectively measured physical function. This is in line with the effect of exercise therapy for each of the chronic conditions investigated (Bartels et al., 2016; Fransen et al., 2015; Puhan et al., 2016; Cooney et al., 2013; Stonerock et al., 2015; McCarthy et al., 2015), and exercise RCTs including people with multimorbidity (de Rooij et al., 2017; Martinez-Velilla et al., 2019). When comparing exercise therapy to primary or community care setting interventions, which include consultations with nurses, physicians and pharmacists aiming at promoting self-management, we observed that for HRQoL and physical function, the improvements promoted by exercise therapy were greater (Salisbury et al., 2018; Smith et al., 2016). However, the indirect comparisons of these interventions, in different multimorbid populations, need to be interpreted with caution.

Exercise therapy appeared to be safe with no risk difference between the exercise therapy and comparator groups on non-serious adverse events. By contrast, exercise therapy reduced the risk for serious adverse events. Although, we were unable to distinguish whether the adverse events were directly related to the interventions, this is promising as adverse events are common in people with chronic conditions (Marengoni and Onder, 2015; Nunes et al., 2016). A systematic review investigating adverse events across all conditions in exercise therapy trials found no risk for an increase of serious adverse events but a 19 % increase in non-serious adverse events (Niemeijer et al., 2019). Differences in risk of non-serious adverse events may be partially explained by the population included and the reduced length of the exercise interventions (on average 13 weeks vs 20 weeks).

### 4.2 Implication for clinical practice

High quality evidence on the effectiveness of different treatments are limited for people with multimorbidity. While future studies are carried out to improve the confidence in the effect of exercise in this population, given that exercise seems safe and beneficial, it can be recommended in clinical practice. People with multimorbidity engaging in an exercise therapy intervention may experience reduced depression and improved health-related quality of life and physical function. These improvements ranged from being not clinically to clinically relevant, with younger people and people with higher depression scores experiencing greater improvements in HRQoL and depression, respectively. For physical function, the improvement observed corresponded to an average increase of 43 m in the 6MWT in favour of the exercise therapy interventions. This improvement is greater than the 30 m cut off for clinical relevance generally used in people with chronic conditions such as chronic obstructive pulmonary disease, lung cancer, coronary artery disease and adults with fear of falling (Bohannon and Crouch, 2017).

### 4.3 Implications for future research

This systematic review highlights a need for future, high quality, RCTs evaluating the effects of well-designed exercise therapy interventions in people with multimorbidity. Based on the result of this systematic review, we suggest that future studies should also include people with different combinations of conditions. For example, osteoarthritis, while being the most common joint disease and often associated with more chronic conditions (Wesseling et al., 2013), has been investigated in only four of the included studies (Hinrichs et al., 2016; Gary et al., 2004; Gretebeck et al., 2019; Leung et al., 2013).

In the design of future exercise interventions, given the fact that multimorbidity is heterogeneous, focusing on individualised treatments tailored to people’s goals and preferences might also help increase the effect estimates and exercise therapy adherence, which is required for expecting greater improvements on health outcomes (Smith et al., 2013; Fransen et al., 2015). In fact, the improvements observed for short-term follow-ups (i.e. immediately after the end of the intervention) were not maintained for depression and anxiety at long-term follow-ups (i.e. the follow-ups closest to 12 months), presumably due to a decrease in exercise adherence. Also, particular attention should be given to the choice and reporting of comparator groups (Freedland et al., 2019). This may help explain the inconsistency of effect estimates. Regarding the selection of the outcome measures, we suggest future studies to select primary outcome measures important to the patients (Smith et al., 2018, 2013), as highlighted by a consensus agreement by a group of international researchers working to improve care for people with multimorbidity.

### 4.4 Strengths and limitations

This systematic review with meta-analysis has been conducted and reported according to recommended international guidelines (Higgins et al., 2019; Moher et al., 2009) and it followed a pre-specified protocol (Bricca et al., 2020). Additionally, we have contacted study authors to retrieve missing information from the included RCTs which allowed us to conduct meta-regression analyses with complete data for intervention characteristics such as mode of delivery and setting of the intervention.

This systematic review has limitations. First, among all the conditions included, the majority of the RCTs included people with depression and heart failure and not all studies included 100 % of people with multimorbidity. Additionally, most people of the included studies reported mild to moderate depression severity, limiting the generalisability of the findings to people with other combinations of conditions or more severe depression. However, the combination of the conditions (studies including participants with heart failure and depression vs. studies including participants with different combination of conditions) and the inclusion criteria (80 % of participants with multimorbidity vs. 100 % with multimorbidity) of this systematic review had no impact on effect estimates. Second, the heterogenous nature of the multimorbidity definition and interventions tested reflected the large inconsistency in the effect estimates of the meta-analyses and that positive results may be attributed to combined effects instead of exercise therapy alone. Third, the quality of evidence for the outcomes of interest was low, suggesting that the true effect of exercise therapy may differ from the one reported in this systematic review. Finally, reporting of adverse events was non-consistent across studies. This is common in exercise trials and highlights the need for harmonising reporting of adverse events (Niemeijer et al., 2019) so that all adverse events are reported regardless of whether they are considered related to the intervention or not.

### 5 Conclusions

While the low quality of the evidence limits the confidence in our results, exercise therapy appeared to be a safe and beneficial intervention to improve physical and psychosocial health in people with multimorbidity, highlighting its potential in the management and care of this population.

## Supplementary Material

Supplementary material related to this article can be found, in the online version, at doi:https://doi.org/10.1016/j.arr.2020.101166.

Supplementary

## Figures and Tables

**Table 1:** Study, participant, intervention and outcome characteristics of the included studies.

Authors, year and study acronym	Country, study design and setting	Condition type and prevalence	Age (mean), gender and BMI (mean)	Intervention characteristics	Duration (minutes), frequency, intensity, length and adherence to the exercise intervention	Comparator characteristics	Outcomes and (outcome measure)	Pre-specified primary outcome
Koukouvou et al. 2004 (39)	Greece, 2-arm RCT, Outpatient fitness centres	D (100 %) HF (100 %) H (12 %)	52 years 0% female BMI 28	Supervised groupbased, aerobic and strengthening exercise	60 min (10 min warm-up and 10 min cool down), 4 times per week for 26 weeks at a moderate intensity. Adherence 78 %.	Usual care	Anxiety (HADS-A) Depression (BDI) HRQoL (QLI)	No protocol found
	USA, 2-arm RCT,	HF (100 %) H (88 %)	68 years				Depression (GDS) HRQoL (MLHFQ)
Gary et al. 2004 (49)	Home-based	KOA (68 %), D (44 %) COPD (34 %) T2DM (31 %)	100 % female BMI 34	Partially supervised self-help aerobic exercise and health education	40 min (5 min warm-up and 5 min cool down), 3 times per week for 12 weeks at a moderate intensity. Adherence 85 %	Health education	PF(6MWT)	No protocol found
Kulcu et al. 2007 (43)	Turkey, 2-arm RCT, Cardiopulmonary rehabilitation clinic	D (100 %) HF (100 %)	59 years 27 % female	Supervised, individual, aerobic exercise	60 min (5 min warm-up and 5 min cool down), 3 times per week for 8 weeks at a moderate intensity 45 min, 3 times per week for 12 weeks at a moderate intensity. Adherence 82 %.	Usual care	Anxiety (TRAIT) Depression (BDI) HRQoL (HQOL)	No protocol found
Gary et al. 2010 (48)	USA, 4-arm RCT, Home-based	D (100 %) HF (100 %) H (88%) T2DM (32 %)	66 years 57 % female	Partially supervised, selfhelp: Aerobic exercise CBT and aerobic exercise, and health education		3) Usual care 4) CBT	Depression (HADS-D) HRQoL (MLHFQ) PF (6MWT)	No protocol found
Åsa et al. 2012 (42)	Sweden, 2-arm RCT, Outpatient Centre-based	D (100 %), T2DM (100 %)	61 years 20 % female BMI 29	Supervised, group-based, aquatic exercise	45 min, 3 times a week for 8 weeks at a low to moderate intensity. Adherence 92 %	Usual care	HRQoL (MLHFQ) PF (6MWT)	No protocol found
Blumenthal et al. 2012 (UPBEAT) (45)	USA, 3-arm RCT	D (100 %) HF (100 %) H (19 %)	64 years 32 % female BMI 31	Supervised, group-based aerobic exercise	30 min (warm up and cool down NR) 3 times per week for 16 weeks at a moderate intensity. Adherence 94 %	Sertraline Placebo pill (up to 200 mg (4 pills)	Depression (HADS-D)	Depression
Blumenthal et al. 2012 (HF-ACTION) (52)	USA, Canada and France, 2-arm RCT, Clinical medical centres	D (100 %) HF (100 %) H (19 %) DM* (10 %)	56 years, 28 % female, BMI 31	Supervised, group-based aerobic exercise	60 min (10 min warm-up and 10 min cool down) 3 times per week for 12 weeks at a moderate intensity. Adherence 41 %	Usual care	Depression (BDI-II)	Composite of all-cause mortality and all-cause hospitalisation rates
Gary et al. 2012 (47)	USA, 2-arm RCT, Home-based	HF (100 %) D (70 %) T2DM (50 %) H (50 %)	60 years 50 % female BMI 34	Partially supervised, self-help aerobic and strengthening exercise and health education	Aerobic exercise: 60 min (10 min warm-up and 10 min cool down) 3 times per week for 12 weeks at a moderate intensity Resistance exercise: 75 min (10 min warm-up and 5 min cool down) 3 times per week for 12 weeks performed at home. Adherence 91 %	Placebo exercise (flexibility and stretching) Usual care	Depression (BDI) HRQoL (KCCQ) PF (6MWT)	No protocol found
Oerkild et al. 2012 (37)	Denmark, 2-arm RCT, Home-based	HF (100 %) H (73 %)	77 years 43 % female COPD (28 %), HRQoL (SF-12pcs) D (18 %)	Self-help aerobic exercise and dietary counselling and, if required, smoking cessation if required	50 min (10 min warm-up and 5 min cool down) 6 times per week for 12 weeks at a low intensity	Usual care	Anxiety (HADS-A) Depression (HADS-D) BMI 27	No protocol found
PF (6MWT)	T2DM (22 %)							
Leung et al. 2013 (53)	AUS, 2-arm RCT, Outpatient pulmonary rehabilitation clinic	COPD (100 %) KOA (60 %) H (55 %) IHD (33 %) T2DM (19 %)	73 years 36 % female BMI 27	Partially supervised, group-based Tai Chi (Sun-style) exercise and DVD for home exercises	60 min (warm up and cool down NR) 2 times per week for 12 weeks at a low intensity performed at a hospital. Adherence 91 %	Usual care	Anxiety (HADS-A) Depression (HADS-D) HRQoL (CRQ) PF (ISWT)	Endurance walking capacity
Nolte et al. 2014 (35) and Edelmann et al. 2011 (34) (Ex-DHF-P)	Germany, 2-arm RCT, Facility-based	HF (100 %) H (82 %) D (64 %) T2DM (14 %)	65 years 56 % female BMI 31	Supervised, individual, aerobic and strengthening exercise	40 min (cycling, 2 times a week) for 4 weeks at a moderate intensity. From week 5 strengthening exercises 2 times per week	Usual care	Depression (PHQ-9) HRQoL (SF-36 pcs) PF (6MWT)	Composite outcome score including are all-cause mortality, hospitalizations, NYHA functional class, global self-rated health, maximal exercise capacity, and diastolic function
Keihani et al 2015 (55)	Iran, 2-arm RCT, institute of cardiovascular rehabilitation in Isfahan	D (100 %) HF (100 %)	61 years 40 % female BMI 29	Supervised, individual, aerobic exercise	60 min (10-15 minutes warm-up, 30-40 minutes aerobic exercises and 10-15 minutes of cooling and relaxing) 3 times per week for 8 weeks at a moderate intensity	Usual care	Anxiety (BDIA) Depression (BDI-D) PF (SF-36 PF)	No protocol found
Pibernik-Okanović et al. 2015 (36)	Croatia, 2-arm RCT, Tertiary diabetes clinic	D (100 %) T2DM (100 %)	66 years 54 % female BMI 30	Supervised, group-based aerobic and strengthening exercise and health education	75 min (warm-up, flexibility, strengthening and stretching exercises) for once a week for 6 weeks	Enhanced usual care BMI 30 Psychoeducation	Depression (CES-D)	Depression
Schneider et al. 2016 (51)	USA, 2-arm RCT, University of Massachusetts Medical School’s	D (100 %) T2DM (100 %)	53 years 100 % female BMI 31	Supervised, group-based, physical activities (walking, Zumba, Pilates, step aerobics, cardiokickboxing, and power yoga) and behavioural activation	90 min (warm up 15 min and cool down 10 min) 2 times per week for 12 weeks at a moderate intensity. Adherence 51 %	Enhanced Usual Care (phone calls to inform participants on their condition)	Depression symptoms (BDI-II)	Depression and HbA1c
Hinrichs et al. 2016 (Homefit) (38)	Germany, 2-arm RCT, Home-based	H (90 %) KOA (60 %) HOA (46 %) T2DM (40 %) HF (33 %) IHD (29 %) COPD (22 %)	80 years 74 % female BMI 31	Partially supervised, selfhelp strength, flexibility, balance and aerobic (walk outside) exercise and physical activity counselling	Aerobic: 30 min 5 days per week for 12 weeks at a moderate intensity Mixed exercises: 3 × 10 or 15 repetitions for strength, flexibility, balance exercises 2 or more per week for 12 weeks. Adherence 84 %	Health education	PF (Chair rise), HRQoL (Medical Outcomes Study 8-item Short-Form Survey)	PF
Bernocchi et al. 2018 (58)	Italy, 2-arm RCT, Home-based	COPD (100 %) D (100 %)	71 years 18 % female BMI 28	Partially supervised, selfhelp, aerobic and callisthenic exercises and health education	Up to 30-45 min of aerobic exercises and up to 30-40 min of muscle reinforcement exercises, up to 7 days a week for 16 weeks at a moderate intensity. Adherence 93 %	Usual Care	HRQoL (MLHFQ), PF (6MWT)	PF
Abdelbasset et al. 2019 (56)	Saudi Arabia, 2-arm RCT, Home-based	D (100 %), HF (100 %) H (20 %)	53 years old, 28 % female, BMI 30	Partially supervised, selfhelp aerobic exercise	60 min, 3 times per week for 12 weeks at a moderate intensity	Usual care	Depression (PHQ-9)	No protocol found
de Groot et al. 2019 (ACTIVE II) (46)	USA, 2-arm RCT, Community fitness centers	D (100 %), T2DM (100 %)	56 years 77 % female	supervised, group-based: Aerobic exercise Aerobic exercise and CBT. All participants received a health education booklet	50 min (10 min warm up and 10 min cool down) 2 times per week for 12 weeks at a moderate intensity	CBT Usual care	Depression (BDI-II) HRQoL (SF-12 pcs)	Depression
Leung et al. 2019 (54)	Hong Kong, 2-arm RCT, Outpatient clinic of a community-based hospital	H (100 %) T2DM (96 %)	64 years old, 48 % female, BMI 27	Supervised, group-based, Tai Chi (Yang-style)	60 min (10 min warm up and 10 min cool down) 2 times per week for 12 weeks at a low intensity. Adherence 81 %	Usual care	HRQoL (SF-12pcs)	No protocol found
Rodriguez-Manas et al. 2019 (MIDFrail) (44)	Europe (Belgium, Czech Republic, United Kingdom, France, Germany, Italy, and Spain), cluster 2-arm RCT, hospital or primary care site	T2DM (100 %) H (100 %) HF (9%)	78 years 49 % female BMI 30	Supervised, individual, strengthening exercise and health and nutritional education	45 min 2 times per week for 18 weeks at a moderate intensity. Adherence 82 %	Usual care	PF (SPPB)	PF
Soliman and Abdelbasset 2019 (57)	Saudi Arabia, 2-arm RCT, Home-based	COPD (100 %), D (100 %) T2DM (100 %)	66 years 20 %female BMI 29	Self-help aerobic exercise	3 times per week for 12 weeks a moderate intensity	Usual care Depression (PHQ-9)	No protocol found
Gretebeck et al. 2019 (50)	USA, 3-arm RCT, Outpatient centrebased	H (83 %) A* (36 %)	71 years 61 % female BMI 33	Supervised group-based: Circuit training and home programme to optimize physical activity Circuit training and health education	50 min (10 min warm up and 10 min cool-down) 3 times per week for 10 weeks at a moderate intensity	Placebo exercise (flexibility and Toning exercises) and health education	PF (6MWT)	PF and physical activity
Campo et al. 2020 (41)	Italy, 2-arm RCT, Home-based	HF (100 %) H (86 %) T2DM (30 %)	77 years 23 % female BMI 27	Partially supervised, self-help aerobic and calisthenic exercise	20 min aerobic exercise plus calisthenic exercises, 3 times per week	Health education	HRQoL (EuroQol-VAS), PF (10 m gait speed)	PF

A*= Arthritis, not specified which form (e.g. osteoarthritis), BDI = Beck depression inventory, BDI-II = Beck depression inventory II, BMI = Body mass index, CES-D = Center for Epidemiologic Studies Depression Scale, COPD = chronic obstructive pulmonary disease, D = Depression, EuroQol-VAS = EQ quality of life visual analogue scale, GDS = Geriatric depression scale, H=Hypertension, HF = heart failure, HADS-D/A=Hospital and anxiety depression scale for depression(D) or anxiety (A), HbA1c= Haemoglobin A1c, HOA=Hip osteoarthritis, HQOL= Hacettepe Quality of Life Questionnaire, HRQoL = health related quality of life, ISWT = Incremental Shuttle Walk Test, MLHFQ = Minnesota Living with Heart Failure Questionnaire, KCCQ = Kansas City Cardiomyopathy Questionnaire, KOA = knee osteoarthritis, PF = physical function, 6MWT = six-minute walking test, RCT = randomised controlled trial, PHQ-9=Patient Health Questionnaire-9, QLI = Quality of Life Index, SF-12 = 12-Item Short Form Health Survey, SF-36 = 36-Item Long Form Health Survey, SPPB = Short Physical Performance Battery), T2DM = type 2 diabetes mellitus, TRAIT = Trait anxiety Inventory.

**Table 2:** Summary of findings.

Outcomes	Risk with Non-exposed comparator groups	Exercise therapy vs. non-exercise therapy Effect size (SMD or MD) with 95 % CI	Relative effect (95 % CI)	N –° of participants (studies)	Certainty of the evidence (GRADE)
Health-Related Quality of Life	-	SMD 0.37 SD higher (0.14 higher to 0.61 higher)	-	967 (13 RCTs)	□□_○○_ LOW ^a,b^
Objectively physical function	-	SMD 0.33 SD higher (0.17 higher to 0.49 higher)	-	2001 (13 RCTs)	□□_○○_ LOW ^a,b^
Self-reported physical function		MD 7.07 SD higher (9.1 lower to 23.23 higher)	-	218 (2 RCTs)	□_○○○_ VERY LOW ^a,b,d^
Depression symptoms	-	SMD 0.8 SD lower (1.21 lower to 0.4 lower)	-	1348 (15 RCTs)	□_○○○_ VERY LOW^b,c,d,e^
Anxiety symptoms	-	SMD 0.49 SD lower (0.99 lower to 0.01 higher)	-	234 (6 RCTs)	□_○○○_ VERY LOW ^a,b^
Non-serious adverse events	154 per 1000	148 per 1000 (82-271)	**RR 0.96** (0.53-1.76)	1620 (12 RCTs)	□□_○○_ LOW ^a,h^
Serious adverse events	172 per 1000	107 per 1000 (85-135)	**RR 0.62** (0.49 to 0.78)	1998 (14 RCTs)	□□_○○_ LOW ^a,b^

**CI:** Confidence interval; **SMD:** Standardised mean difference; **MD:** Mean difference; **RR:** Risk ratio. **GRADE Working Group grades of evidence High certainty:** We are very confident that the true effect lies close to that of the estimate of the effect **Moderate certainty:** We are moderately confident in the effect estimate: The true effect is likely to be close to the estimate of the effect, but there is a possibility that it is substantially different **Low certainty:** Our confidence in the effect estimate is limited: The true effect may be substantially different from the estimate of the effect **Very low certainty:** We have very little confidence in the effect estimate: The true effect is likely to be substantially different from the estimate of effect. Explanations.

a Quality of evidence downgraded of one level for indirectness of the population.

b Quality of evidence downgraded of one level for imprecision of the estimates.

c Quality of evidence downgraded of one level for inconsistency of the estimates.

d Quality of evidence downgraded of one level due to risk of bias.

e Publication bias strongly suspected.

## References

[R1] Abdelbasset WK, Alqahtani BA, Alrawaili SM, Ahmed AS, Elnegamy TE, Ibrahim AA (2019). Similar effects of low to moderate-intensity exercise program vs moderate-intensity continuous exercise program on depressive disorder in heart failure patients A 12-week randomized controlled trial. Medicine.

[R2] Anon (2018). The Lancet - making more of multimorbidity: an emerging priority. Lancet.

[R3] Anon (2020). FDA Serious Adverse Event. https://www.fda.gov/safety/reporting-serious-problems-fda/what-serious-adverse-event.

[R4] Åsa C, Maria S, Katharina SS, Bert A (2012). Aquatic exercise is effective in improving exercise performance in patients with heart failure and type 2 diabetes mellitus. Evid-Based Compl Altern.

[R5] Bartels EM, Juhl CB, Christensen R (2016). Aquatic exercise for the treatment of knee and hip osteoarthritis. Cochrane Database Syst Rev.

[R6] Bayliss EA, Bayliss MS, Ware JE, Steiner JF (2004). Predicting declines in physical function in persons with multiple chronic medical conditions: what we can learn from the medical problem list. Health Qual Life Outcomes.

[R7] Bernocchi P, Vitacca M, La Rovere MT, Volterrani M, Galli T, Baratti D (2018a). Home-based telerehabilitation in older patients with chronic obstructive pulmonary disease and heart failure: a randomised controlled trial. Age Ageing.

[R8] Bernocchi P, Vitacca M, La Rovere MT, Volterrani M, Galli T, Baratti D (2018b). Home-based telerehabilitation in older patients with chronic obstructive pulmonary disease and heart failure: a randomised controlled trial. Age Ageing.

[R9] Blumenthal JA, Sherwood A, Babyak MA, Watkins LL, Smith PJ, Hoffman BM (2012a). Exercise and pharmacological treatment of depressive symptoms in patients with coronary heart disease: results from the UPBEAT (understanding the Prognostic Benefits of Exercise and Antidepressant Therapy) study. J Am Coll Cardiol.

[R10] Blumenthal JA, Babyak MA, O’Connor C, Keteyian S, Landzberg J, Howlett J (2012b). Effects of exercise training on depressive symptoms in patients with chronic heart failure: the HF-ACTION randomized trial. JAMA.

[R11] Bohannon RW, Crouch R (2017). Minimal clinically important difference for change in 6-minute walk test distance of adults with pathology: a systematic review. J Eval Clin Pract.

[R12] Bricca A, Harris KL, Saracutu M, Smith MS, Juhl BJ, Skou ST (2020). The benefits and harms of therapeutic exercise on physical and psychosocial outcomes in people with multimorbidity: protocol for a systematic review. J Comorbidity.

[R13] Campo G, Tonet E, Chiaranda G, Sella G, Maietti E, Bugani G (2020). Exercise intervention improves quality of life in older adults after myocardial infarction: randomised clinical trial. Heart.

[R14] Caspersen CJ, Powell KE, Christenson GM (1985). Physical-activity, exercise, and physical-fitness - definitions and distinctions for health-related research. Public Health Rep.

[R15] Cohen J (1988). Statistical power analysis for the behavioral-sciences. Percept Motor Skill.

[R16] Cooney GM, Dwan K, Greig CA, Lawlor DA, Rimer J, Waugh FR (2013). Exercise for depression. Cochrane Database Syst Rev.

[R17] Cornelissen VA, Smart NA (2013). Exercise training for blood pressure: a systematic review and meta-analysis. J Am Heart Assoc.

[R18] de Groot M, Shubrook JH, Hornsby WG, Pillay Y, Mather KJ, Fitzpatrick K (2019). Program ACTIVE II: outcomes from a randomized, multistate community-based depression treatment for rural and urban adults with type 2 diabetes. Diabetes Care.

[R19] de Rooij M, van der Leeden M, Cheung J, van der Esch M, Hakkinen A, Haverkamp D (2017). Efficacy of tailored exercise therapy on physical functioning in patients with knee osteoarthritis and comorbidity: a randomized controlled trial. Arthritis Care Res (Hoboken).

[R20] Edelmann F, Gelbrich G, Dungen HD, Frohling S, Wachter R, Stahrenberg R (2011). Exercise training improves exercise capacity and diastolic function in patients with heart failure with preserved ejection fraction results of the Ex-DHF (Exercise training in diastolic heart failure) pilot study. J Am Coll Cardiol.

[R21] Fortin M, Lapointe L, Hudon C, Vanasse A, Ntetu AL, Maltais D (2004). Multimorbidity and quality of life in primary care: a systematic review. Health Qual Life Outcomes.

[R22] Fortin M, Bravo G, Hudon C, Lapointe L, Dubois MF, Almirall J (2006). Psychological distress and multimorbidity in primary care. Ann Fam Med.

[R23] Foster ED, Deardorff A (2017). Open science framework (OSF). J Med Libr Assoc.

[R24] Fransen M, McConnell S, Harmer AR, Van der Esch M, Simic M, Bennell KL (2015). Exercise for osteoarthritis of the knee. Cochrane Database Syst Rev.

[R25] Freedland KE, King AC, Ambrosius WT, Mayo-Wilson E, Mohr DC, Czajkowski SM (2019). The selection of comparators for randomized controlled trials of health-related behavioral interventions: recommendations of an NIH expert panel. J Clin Epidemiol.

[R26] Gary RA, Sueta CA, Dougherty M, Rosenberg B, Cheek D, Preisser J (2004). Home-based exercise improves functional performance and quality of life in women with diastolic heart failure. Heart Lung.

[R27] Gary RA, Dunbar SB, Higgins MK, Musselman DL, Smith AL (2010). Combined exercise and cognitive behavioral therapy improves outcomes in patients with heart failure. J Psychosom Res.

[R28] Gary RA, Cress ME, Higgins MK, Smith AL, Dunbar SB (2012). A Combined Aerobic and Resistance Exercise Program Improves Physical Functional Performance in Patients With Heart Failure A Pilot Study. J Cardiovasc Nurs.

[R29] Gleeson M, Bishop NC, Stensel DJ, Lindley MR, Mastana SS, Nimmo MA (2011). The anti-inflammatory effects of exercise: mechanisms and implications for the prevention and treatment of disease. Nat Rev Immunol.

[R30] Global Burden of Disease Study 2013 Collaborators (2015). Global, regional, and national incidence, prevalence, and years lived with disability for 301 acute and chronic diseases and injuries in 188 countries, 1990-2013: a systematic analysis for the Global Burden of Disease Study 2013. Lancet.

[R31] Gretebeck KA, Blaum CS, Moore T, Brown R, Galecki A, Strasburg D (2019). Functional exercise improves mobility performance in older adults with type 2 diabetes: a randomized controlled trial. J Phys Act Health.

[R32] Higgins JPTTJ, Chandler J, Cumpston M, Li T, Page MJ, Welch VA (2019). Cochrane Handbook for Systematic Reviews of Interventions Version 6.0 (Updated July 2019). Cochrane.

[R33] Hinrichs T, Bücker B, Klaaßen-Mielke R, Brach M, Wilm S, Platen P (2016). Home-based exercise supported by general practitioner practices: ineffective in a sample of chronically ill, mobility-limited older adults (the HOMEfit randomized controlled trial). J Am Geriatr Soc.

[R34] Keihani DK, Mokhtari M, Mokhtari M (2014). Cardiac effects of exercise rehabilitation on quality of life,depression and anxiety in patients with heart failure patients. J Fundam Mental Health.

[R35] Koukouvou G, Kouidi E, Iacovides A, Konstantinidou E, Kaprinis G, Deligiannis A (2004). Quality of life, psychological and physiological changes following exercise training in patients with chronic heart failure. J Rehabil Med.

[R36] Kulcu DG, Kurtais Y, Tur BS, Gulec S, Seckin B (2007). The effect of cardiac rehabilitation on quality of life, anxiety and depression in patients with congestive heart failure. A randomized controlled trial, short-term results. Medicophys.

[R37] Leung RW, McKeough ZJ, Peters MJ, Alison JA (2013). Short-form Sun-style t’ai chi as an exercise training modality in people with COPD. Eur Respir J.

[R38] Leung L-L, Chan A-K, Sit J-H, Liu T, Taylor-Piliae RE (2019). Tai Chi in Chinese adults with metabolic syndrome: a pilot randomized controlled trial. Complement Ther Med.

[R39] Long L, Mordi IR, Bridges C, Sagar VA, Davies EJ, Coats AJ (2019). Exercise-based cardiac rehabilitation for adults with heart failure. Cochrane Database Syst Rev.

[R40] Marengoni A, Onder G (2015). Guidelines, polypharmacy, and drug-drug interactions in patients with multimorbidity. BMJ.

[R41] Martinez-Velilla N, Casas-Herrero A, Zambom-Ferraresi F, de Asteasu MLS, Lucia A, Galbete A (2019). Effect of exercise intervention on functional decline in very elderly patients during acute hospitalization a randomized clinical trial. JAMA Intern Med.

[R42] McCarthy B, Casey D, Devane D, Murphy K, Murphy E, Lacasse Y (2015). Pulmonary rehabilitation for chronic obstructive pulmonary disease. Cochrane Database Syst Rev.

[R43] Menotti A, Mulder I, Nissinen A, Giampaoli S, Feskens EJM, Kromhout D (2001). Prevalence of morbidity and multimorbidity in elderly male populations and their impact on 10-year all-cause mortality: the FINE study (Finland, Italy, Netherlands, Elderly). J Clin Epidemiol.

[R44] Moher D, Liberati A, Tetzlaff J, Altman DG, Group P (2009). Preferred reporting items for systematic reviews and meta-analyses: the PRISMA statement. BMJ.

[R45] Muth C, Blom JW, Smith SM, Johnell K, Gonzalez-Gonzalez AI, Nguyen TS (2019). Evidence supporting the best clinical management of patients with multimorbidity and polypharmacy: a systematic guideline review and expert consensus. J Intern Med.

[R46] National Institute for Health and Care Excellence (2013). Physical Activity: Brief Advice for Adults in Primary Care. Public Health Guideline [PH44].

[R47] Niemeijer A, Lund H, Stafne SN, Ipsen T, Goldschmidt CL, Jorgensen CT (2019). Adverse events of exercise therapy in randomised controlled trials: a systematic review and meta-analysis. Br J Sports Med.

[R48] Nolte K, Herrmann-Lingen C, Wachter R, Gelbrich G, Dungen HD, Duvinage A (2015). Effects of exercise training on different quality of life dimensions in heart failure with preserved ejection fraction: the Ex-DHF-P trial. Eur J Prev Cardiol.

[R49] Nunes BP, Flores TR, Mielke GI, Thume E, Facchini LA (2016). Multimorbidity and mortality in older adults: a systematic review and meta-analysis. Arch Gerontol Geriatr.

[R50] Oerkild B, Frederiksen M, Hansen JF, Prescott E (2012). Home-based cardiac rehabilitation is an attractive alternative to no cardiac rehabilitation for elderly patients with coronary heart disease: results from a randomised clinical trial. BMJ Open.

[R51] Parker SG, Corner L, Laing K, Nestor G, Craig D, Collerton J (2019). Priorities for research in multiple conditions in later life (multi-morbidity): findings from a James Lind Alliance Priority Setting Partnership. Age Ageing.

[R52] Pedersen BK, Saltin B (2015). Exercise as medicine - evidence for prescribing exercise as therapy in 26 different chronic diseases. Scand J Med Sci Sport.

[R53] Pefoyo AJ, Bronskill SE, Gruneir A, Calzavara A, Thavorn K, Petrosyan Y (2015). The increasing burden and complexity of multimorbidity. BMC Public Health.

[R54] Pibernik-Okanovic M, Hermanns N, Ajdukovic D, Kos J, Prasek M, Sekerija M (2015). Does treatment of subsyndromal depression improve depression-related and diabetes-related outcomes? A randomised controlled comparison of psychoeducation, physical exercise and enhanced treatment as usual. Trials.

[R55] Puhan MA, Gimeno-Santos E, Cates CJ, Troosters T (2016). Pulmonary rehabilitation following exacerbations of chronic obstructive pulmonary disease. Cochrane Database Syst Rev.

[R56] Reeuwijk KG, de Rooij M, van Dijk GM, Veenhof C, Steultjens MP, Dekker J (2010). Osteoarthritis of the hip or knee: which coexisting disorders are disabling?. Clin Rheumatol.

[R57] Rodriguez-Manas L, Laosa O, Vellas B, Paolisso G, Topinkova E, Oliva-Moreno J (2019). Effectiveness of a multimodal intervention in functionally impaired older people with type 2 diabetes mellitus. J Cachexia Sarcopenia Muscle.

[R58] Salisbury C, Man MS, Bower P, Guthrie B, Chaplin K, Gaunt DM (2018). Management of multimorbidity using a patient-centred care model: a pragmatic cluster-randomised trial of the 3D approach. Lancet.

[R59] Schneider KL, Panza E, Handschin B, Ma Y, Busch AM, Waring ME (2016). Feasibility of pairing behavioral activation with exercise for women with type 2 diabetes and depression: the get it study pilot randomized controlled trial. Behav Ther.

[R60] Schünemann HBJ, Guyatt G, Oxman A (2013). GRADE Handbook for Grading Quality of Evidence and Strength of Recommendations. The GRADE Working Group.

[R61] Sinnott C, Mc Hugh S, Browne J, Bradley C (2013). GPs’ perspectives on the management of patients with multimorbidity: systematic review and synthesis of qualitative research. BMJ Open.

[R62] Smith SM, Bayliss EA, Mercer SW, Gunn J, Vestergaard M, Wyke S (2013). How to design and evaluate interventions to improve outcomes for patients with multimorbidity. J Comorb.

[R63] Smith SM, Wallace E, O’Dowd T, Fortin M (2016). Interventions for improving outcomes in patients with multimorbidity in primary care and community settings. Cochrane Database Syst Rev.

[R64] Smith SM, Wallace E, Salisbury C, Sasseville M, Bayliss E, Fortin M (2018). A core outcome set for multimorbidity research (COSmm). Ann Fam Med.

[R65] Soliman GS, Abdelbasset WK (2019). Efficacy of aerobic training on pulmonary functions and depression in elderly COPD patients. J Adv Pharm Educ Res.

[R66] Stonerock GL, Hoffman BM, Smith PJ, Blumenthal JA (2015). Exercise as treatment for anxiety: systematic review and analysis. Ann Behav Med.

[R67] Vogeli C, Shields AE, Lee TA, Gibson TB, Marder WD, Weiss KB (2007). Multiple chronic conditions: prevalence, health consequences, and implications for quality, care management, and costs. J Gen Intern Med.

[R68] Wesseling J, Welsing PMJ, Bierma-Zeinstra SMA, Dekker J, Gorter KJ, Kloppenburg M (2013). Impact of self-reported comorbidity on physical and mental health status in early symptomatic osteoarthritis: the CHECK (Cohort Hip and Cohort Knee) study. Rheumatology.

