# Supplementary material for: Benefits and harms of exercise therapy in people with multimorbidity: A systematic review and meta-analysis of randomised controlled trials
Source: Ageing Res Rev. Author manuscript; Available in PMC 2020 Sep 25. (PMC7116122; doi:10.1016/j.arr.2020.101166)
Supplement: Supplementary [file EMS94911-supplement-Supplementary.zip › 1-s2.0-S1568163720303019-mmc1.docx]

**Supplementary table 1. Impact of study level covariates on effect estimates.**

|  | **Health-related quality of life** | | | | | **Physical function objectively measured** | | | | | | **Depression** | | | | | | |  |
| --- | --- | --- | --- | --- | --- | --- | --- | --- | --- | --- | --- | --- | --- | --- | --- | --- | --- | --- | --- |
|  | **Number of comparisons** | **Effect size (SMD)** | **95%CI low** | **95%CI high** | **tau2** | **Number of comparisons** | **Effect size (SMD)** | **95%CI low** | **95%CI high** | **tau2** | **Number of comparisons** | | **Effect size (SMD)** | **95%CI low** | **95%CI high** | **tau2** | |  |  |
|  | 15 | 0.37 | 0.14 | 0.61 | 0.11 | 16 | 0.33 | 0.17 | 0.49 | 0.04 | 19 | | -0.8 | -1.21 | -0.4 | 0.7 | |  |  |
|  | **Number of comparisons** | **Coef.** | **95%CI low** | **95%CI high** | **tau2** | **Number of comparisons** | **Coef.** | **95%CI low** | **95%CI high** | **tau2** | **Number of comparisons** | | **Coef.** | **95%CI low** | **95%CI high** | **tau2** | |  |  |
| **PARTICIPANT CHARACTERISTICS** | |  |  |  |  |  |  |  |  |  | |  |  |  |  |  | | | |
| **Age** | 15 | -0.03 | -0.05 | -0.01 | 0.09 | 16 | -0.01 | -0.03 | 0.01 | 0.04 | 19 | | 0.05 | -0.01 | 0.1 | 0.63 | |  |  |
| **Female proportion** | 15 | -0.01 | -0.15 | 0.01 | 0.10 | 16 | -0.01 | -0.02 | 0.01 | 0.02 | 19 | | 0.01 | -0.01 | 0.03 | 0.66 | |  |  |
| **BMI** | 10 | 0.01 | -0.13 | 0.15 | 0.21 | 12 | -0.01 | -0.09 | 0.07 | 0.05 | 14 | | 0.09 | -0.12 | 0.3 | 1.03 | |  |  |
| **Low Socio Economic Status** | 10 | -0.01 | -0.54 | 0.54 | 0.06 | 11 | 0.55 | -0.36 | 1.47 | 0.04 | 13 | | 0.09 | -1.44 | 1.63 | 0.91 | |  |  |
| **Hypertension proportion** | 10 | -0.01 | -0.03 | -0.01 | 0.07 | 12 | -0.01 | -0.02 | 0.01 | 0.04 | 10 | | 0.01 | -0.01 | 0.01 | 0.01 | |  |  |
| **Type 2 diabetes proportion** | 11 | 0.01 | -0.10 | 0.10 | 0.05 | 14 | -0.01 | -0.01 | 0.01 | 0.05 | NA | | | | | |  |  |  |
| **Depression severity** | 10 | 0.01 | -0.05 | 0.05 | 0.26 | NA | | | | | 18 | | -0.04 | -0.7 | -0.02 | 0.31 | |  |  |
| **Heart failure proportion** | 11 | 0.01 | -0.1 | 0.02 | 0.12 | 11 | 0.01 | 0.01 | 0.01 | 0.01 | NA | | | | | |  |  |  |
| **Multimorbidity**  **(100% vs. 80%)** | 15 | -0.30 | -0.75 | 0.15 | 0.11 | 16 | -2.23 | -5.75 | 0.11 | 0.04 | 19 | | 0.58 | -0.41 | 1.58 | 0.69 | |  |  |
| **INTERVENTION CHARACTERISTICS** | |  |  |  |  |  |  |  |  |  | |  |  |  |  |  | | | |
| **Type of intervention** |  |  |  |  |  |  |  |  |  |  |  | |  |  |  |  | |  |  |
| Mixed vs Aerobic | 15 | -0.41 | -0.93 | 0.1 | 0.15 | 16 | -0.01 | -0.39 | 0.38 | 0.04 | 19 | | -0.31 | -1.36 | 0.74 | 0.74 | |  |  |
| Mixed vs Resistance | NA | | | | | 16 | -0.03 | -0.07 | 0.14 | 0.04 | NA | | | | | |  |  |  |
| Mixed vs Tai Chi | 15 | -0.36 | -1.13 | 0.41 | 0.15 | 16 | -0.41 | -1.15 | 0.35 | 0.04 | 19 | | 0.31 | -1.7 | 2.33 | 0.74 | |  |  |
| Frequency (sessions per week) | 15 | -0.02 | -0.17 | 0.14 | 0.13 | 16 | 0.05 | -0.06 | 0.15 | 0.04 | 19 | | 0.04 | -0.39 | 0.47 | 0.75 | |  |  |
| **Intervention setting** |  |  |  |  |  |  |  |  |  |  |  | |  |  |  |  | |  |  |
| Hospital vs home | 15 | -0.14 | -0.81 | 0.53 | 0.10 | 16 | 0.44 | -0.29 | 1.17 | 0.03 | 19 | | -0.64 | -2.11 | 0.83 | 0.79 | |  |  |
| Hospital vs outpatient | 15 | 0.30 | -0.43 | 1.1 | 0.10 | 16 | 0.19 | -0.54 | 0.93 | 0.03 | 19 | | 0.02 | -1.47 | 1.5 | 0.79 | |  |  |
| **Mode of delivery** |  |  |  |  |  |  |  |  |  |  |  | |  |  |  |  | |  |  |
| Group vs individual | 15 | -0.40 | -1.36 | 0.54 | 0.11 | 16 | -0.17 | -0.6 | 0.29 | 0.03 | 19 | | -0.54 | -1.85 | 0.76 | 0.64 | |  |  |
| Group vs self-help | 15 | -0.41 | -0.9 | 0.07 | 0.11 | 16 | 0.19 | -0.16 | 0.55 | 0.03 | 19 | | -0.78 | -1.61 | 0.06 | 0.64 | |  |  |
| **Adherence to exercise intervention** | 10 | 0.02 | -0.05 | 0.08 | 0.15 | NA | | | | | 11 | | -0.01 | -0.02 | 0.01 | 1.8 | |  |  |
| **Intervention length (in weeks)** | 15 | 0.05 | 0.02 | 0.09 | 0.09 | 16 | 0.01 | -0.03 | 0.05 | 0.5 | 19 | | -0.01 | -0.13 | 0.09 | 0.75 | |  |  |
| **Supervision** | 15 | 0.42 | -0.23 | 1.07 | 0.12 | 16 | -0.19 | -0.54 | 0.15 | 0.04 | 19 | | 0.24 | -0.63 | 1.11 | 0.75 | |  |  |
| **RISK OF BIAS** |  |  |  |  |  |  |  |  |  |  |  | |  |  |  |  | |  |  |
| **Low vs Some concerns or high** | 15 | -0.15 | -0.64 | 0.33 | 0.13 | 16 | 0.33 | 0.03 | 0.64 | 0.03 | 19 | | -0.79 | -1.56 | -0.03 | 0.57 | |  |  |

NA=Not applicable, covariates with less than 10 studies provided outcomes for the covariates of interest.
