# Supplementary material for: Benefits and harms of exercise therapy in people with multimorbidity: A systematic review and meta-analysis of randomised controlled trials
Source: Ageing Res Rev. Author manuscript; Available in PMC 2020 Sep 25. (PMC7116122; doi:10.1016/j.arr.2020.101166)
Supplement: Supplementary [file EMS94911-supplement-Supplementary.zip › 1-s2.0-S1568163720303019-mmc4.docx]

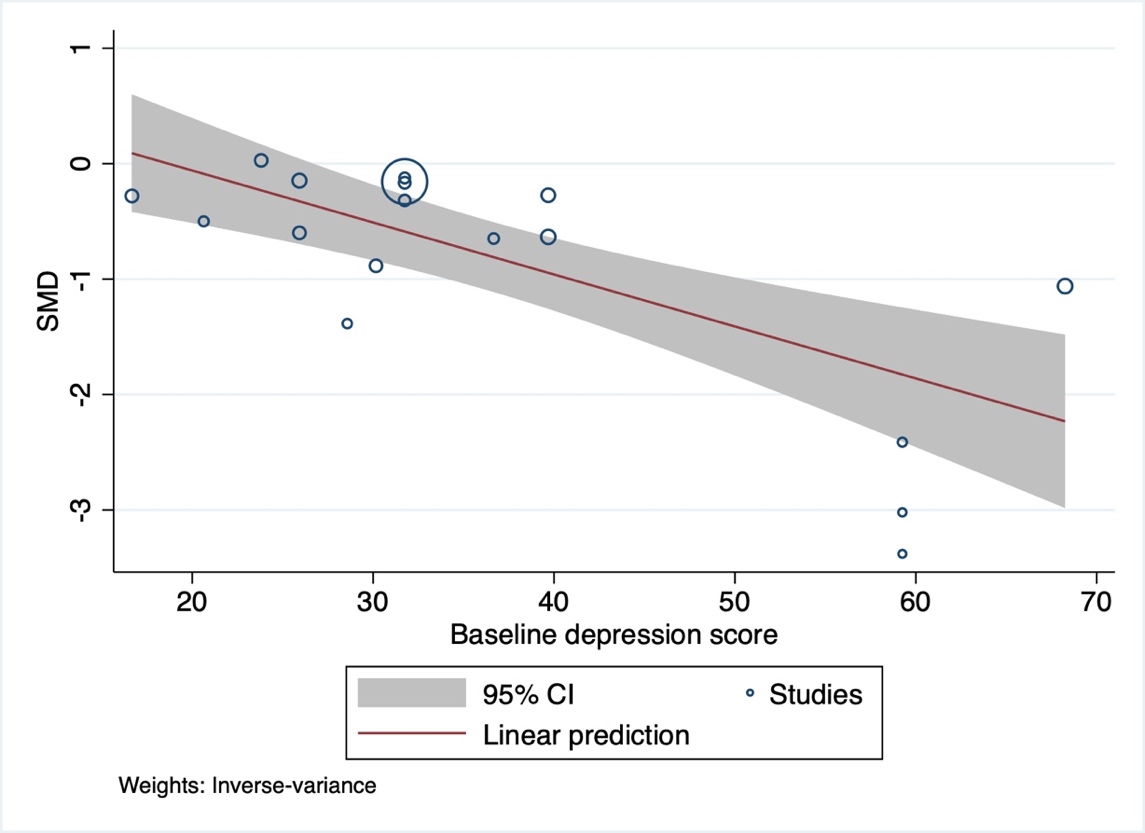


**Supplementary 3.** Bubble plot showing the meta-regression analysis of the studies included in the meta-analysis for the effect of exercise therapy on depression. SMDs reduction of depression symptoms (y-axis) and baseline depression score (x-axis) are shown. Higher scores represent higher depression at baseline. Weights of included trials were based on the inverse variance and are shown by the size of the circles (a smaller circle contributes less to the overall result).
