# Supplementary material for: Benefits and harms of exercise therapy in people with multimorbidity: A systematic review and meta-analysis of randomised controlled trials
Source: Ageing Res Rev. Author manuscript; Available in PMC 2020 Sep 25. (PMC7116122; doi:10.1016/j.arr.2020.101166)
Supplement: Supplementary [file EMS94911-supplement-Supplementary.zip › 1-s2.0-S1568163720303019-mmc6.docx]

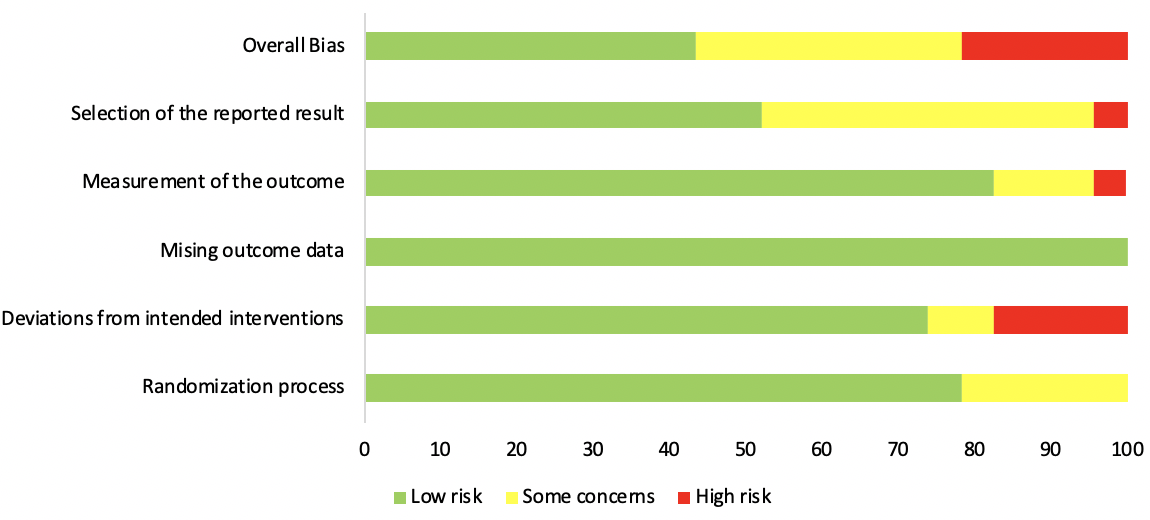


**Supplementary figure 5. ‘**Risk of bias’ summary of 23 individual ‘Risk of bias’ items for each included study.
