# Supplementary material for: Benefits and harms of exercise therapy in people with multimorbidity: A systematic review and meta-analysis of randomised controlled trials
Source: Ageing Res Rev. Author manuscript; Available in PMC 2020 Sep 25. (PMC7116122; doi:10.1016/j.arr.2020.101166)
Supplement: Supplementary [file EMS94911-supplement-Supplementary.zip › 1-s2.0-S1568163720303019-mmc7.docx]

a)
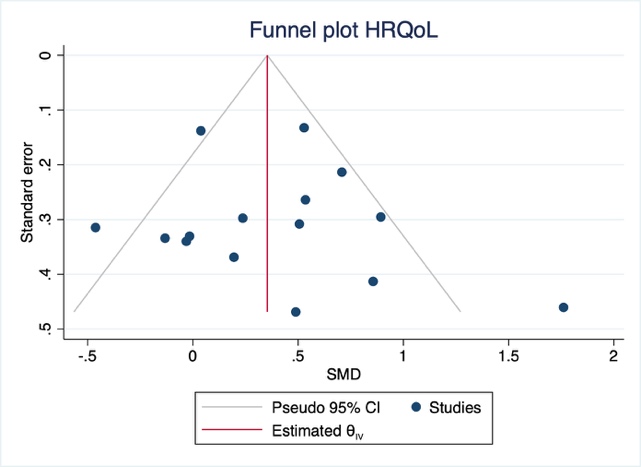
 b)
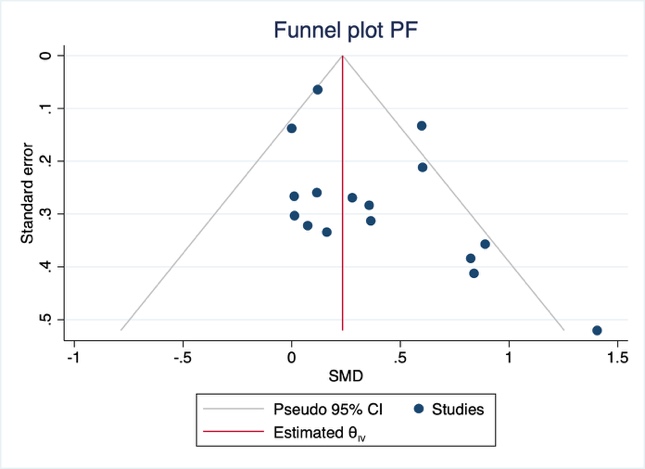


c)
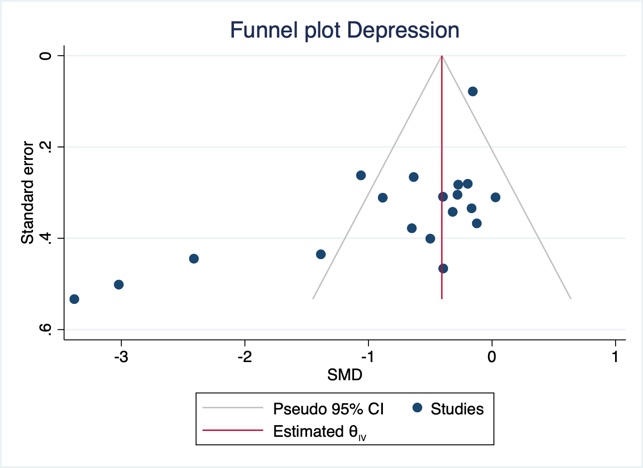


**Supplementary figure 6:** Funnel plots investigating small study bias for the outcomes Health-related quality of life (a), objectively measured physical function (b) and depression symptoms (c)
