# Supplementary material for: Benefits and harms of exercise therapy in people with multimorbidity: A systematic review and meta-analysis of randomised controlled trials
Source: Ageing Res Rev. Author manuscript; Available in PMC 2020 Sep 25. (PMC7116122; doi:10.1016/j.arr.2020.101166)
Supplement: Supplementary [file EMS94911-supplement-Supplementary.zip › 1-s2.0-S1568163720303019-mmc3.docx]

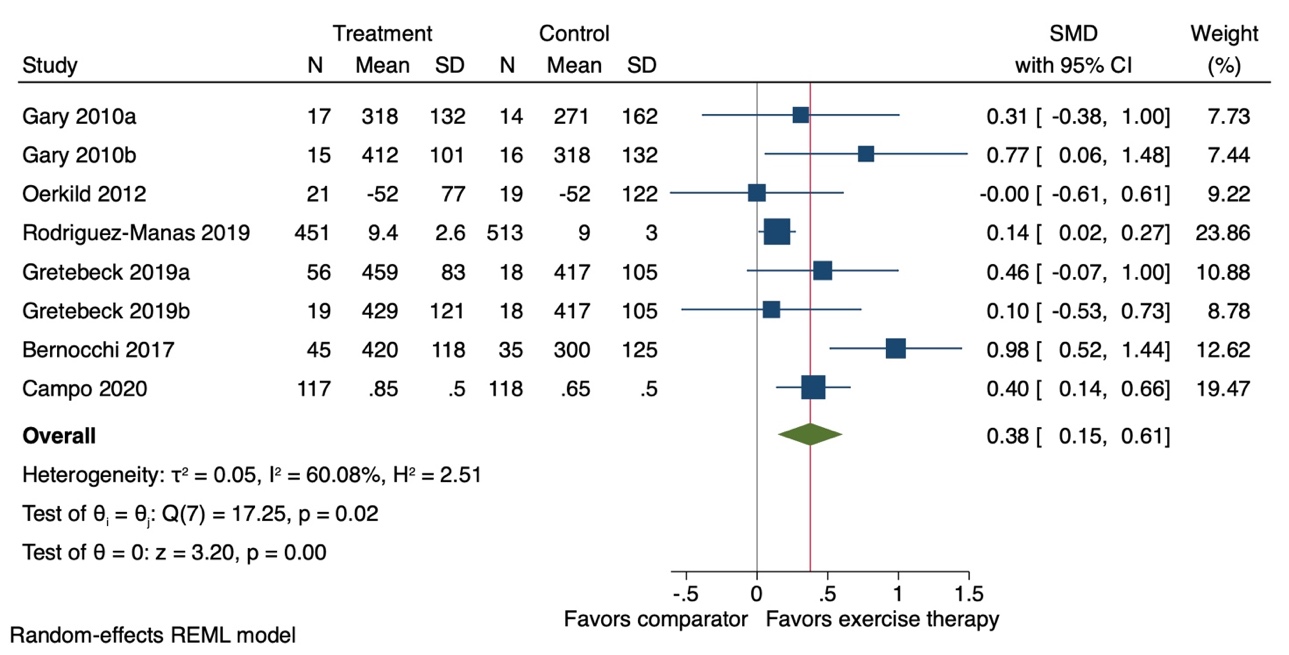


**Supplementary Figure 2**. Forest plot for the long-term effect (follow-up closest to 12-month post randomisation, mean 33.5 weeks (SD 15.4)) of exercise therapy compared to a non-exercise therapy comparator group on objectively measured physical function. SMD=Standardised Mean Difference.
